# The predictive value of the urinary iodine-to-creatine ratio in spot samples for the estimation of 24-hour urinary iodine excretion in adult non-pregnant women

**DOI:** 10.3389/fendo.2025.1727103

**Published:** 2025-12-10

**Authors:** Jerzy Chudek, Joanna Fryżewska, Piotr Kocełak, Aleksander J. Owczarek, Piotr Mikuła, Anna Chudek, Joanna Staniszewska-Mróz, Mateusz Winder, Magdalena Olszanecka-Glinianowicz, Gabriela A. Handzlik

**Affiliations:** 1 Department of Internal Medicine and Oncological Chemotherapy, Medical University of Silesia, Katowice, Poland; 2 Health Promotion and Obesity Management Unit, Department of Pathophysiology, Medical University of Silesia in Katowice, Katowice, Poland; 3 Omni Analytics Poland, Łódź, Poland; 4 Department of Radiology and Nuclear Medicine, Medical University of Silesia, Katowice, Poland

**Keywords:** iodine excretion, 24-hour urine collection, iodine-to-creatinine ratio, thyroid function, urine spot sample, iodine deficiency

## Abstract

**Objective:**

The study aimed to compare iodine-to-creatinine ratios in morning spot samples with 24-hour iodine excretion, serving as the reference standard in adult non-pregnant women. The additional objective was to examine the accuracy of the iodine-to-creatinine ratio in morning spot samples for assessing iodine intake.

**Methods:**

A cross-sectional, observational study included 49 non-pregnant adult women undergoing endocrine evaluation for adrenal incidentalomas in a hospital setting. Iodine and creatinine concentrations were measured in 24-hour collections and morning spot urine samples. The cut-off value for iodine deficiency in the 24-hour iodine excretion, based on iodine/creatinine in the urine spot sample, was assessed based on the ROC curve analysis.

**Results:**

The iodine-to-creatinine ratio in a morning spot urine sample correlated significantly with 24-hour urinary iodine excretion (R = 0.196; p = 0.002), unlike iodine concentration in the urine spot sample. Sufficient urinary iodine excretion (≥150 µg/day) was found in only 16.3% of participants, while iodine deficiency (<100 µg/day) was observed in 55.1%. The iodine-to-creatinine ratio cut-off of 73 µg/g identified iodine deficiency with 66.7% sensitivity and 72.7% specificity. However, its ability to detect mild insufficiency was limited. All cases of moderate deficiency were correctly classified.

**Conclusions:**

Iodine to creatine ratio in morning spot urine sample modestly reflects 24-hour iodine urinary excretion but more accurately that iodine concentration alone. A cut-off value of 73 μg/g offers moderate diagnostic performance. A daily iodine intake of 100 µg may be sufficient to prevent its deficiency, suggesting reconsideration of current recommendations for iodine intake.

## Introduction

Iodine has a significant impact on thyroid function, fetal development, and the process of thyroid carcinogenesis (1, 2). Iodine deficiencies observed worldwide are mainly considered low-to-moderate, while iodine excess is reported in only thirteen countries. Thus, iodine deficiency remains a problem despite the implementation of programs aimed at addressing it. A main approach to balancing insufficient iodine content in the diet is iodization of edible salt. However, this makes iodine intake dependent on the individual diet and salt consumption. Since urinary iodine excretion (UIE) is considered constant, the most accurate way to measure iodine resources in the body is through a spot or 24-hour urine collection, for population and individual purposes, respectively (3, 4). The population standards for urinary iodine concentration (UIC) in teenagers and adults proposed by the World Health Organization (WHO) range from 100 to 299 µg/L (150-249 µg/L in pregnant women and ≥100 µg/L in breastfeeding women and children less than 2 years) (5). UIC corresponds to iodine intake over the last few days or even hours, making spot UIC analyses highly variable (6, 7). This may be a cause of a falsely overestimated incidence of iodine deficiency, especially when the first spot sample is collected in the morning (8, 9). On the other hand, a 24-hour urine collection is prone to errors due to the inconvenience for patients and the dependence of iodine concentration on overall urine volume (10). Thus, an estimated UIE from spot samples is used based on the age and sex-adjusted urine iodine to creatinine ratio (UI/Cr). Adjusting the ratio is necessary due to differences in urinary creatinine excretion (11, 12). The UI/Cr was found to be similar to serum iodine levels and to reflect 24-hour urinary iodine excretion better than concentrations in spot samples in pregnant and postpartum women (8). The level of agreement between correct assignments to groups with sufficient and insufficient iodine, as determined by spot UIC and UI/Cr, reaches 84% (13). The UIC, UI/Cr, and estimated 24-hour UIE values from a spot urine sample correlate with each other as shown in the adult Japanese population (14). However, the estimation method may require as many as ten spot urine samples to assess the individual iodine status with similar precision to a single 24-hour urine collection (15). The time of sampling also affects the reliability of measurements. The estimated 24-hour UI/Cr based on the second-morning sample and the last sample collected in the evening before going to bed does not differ significantly from the actual 24-hour UIE, contrary to the estimated 24-hour UI/Cr from the first-morning urine sample (9).

The primary objective of our study was to compare iodine-to-creatinine ratios in morning spot samples with 24-hour UIE, serving as the reference standard in adult non-pregnant women. The secondary objective was to estimate the accuracy of the iodine-to-creatinine ratio in morning spot samples for assessing iodine intake.

## Materials and methods

### Study design

This was a cross-sectional, observational study conducted on inpatients of the Endocrinology Unit of the Department of Internal Medicine and Oncological Chemotherapy, Katowice, Poland, designed to evaluate the predictive accuracy of spot urine-based methods for estimating 24-hour UIE. We chose the morning spot urinary sample based on current recommendations of the World Health Organization (16). Adult female patients undergoing endocrine evaluation for adrenal incidentalomas during short hospitalization with urine collections were enrolled in this study.

Participants were considered eligible for inclusion if they provided written informed consent and were at least 18 years old. Exclusion criteria included adherence to restrictive or elimination diets (e.g., vegan, vegetarian, dairy-free, or gluten-free), diagnosis or treatment of thyroid disorders, a history of total or partial thyroidectomy, recent administration of therapeutic or diagnostic iodine (iodine-131 within the past 12 months or iodine-123 within the past 6 months), use of iodine-containing medications and supplements (e.g., levothyroxine preparations, seaweed extracts), exposure to iodine-based contrast agents within the preceding 3 months, treatment with amiodarone, use of iodine-containing ophthalmic solutions or use of iodine-containing antiseptic agents.

The Bioethics Committee of the Medical University of Silesia in Katowice granted ethical approval (PCN/CBN/0052/KB1/77/I/22 from 20 Sep 2022).

### Patient population

The study included 49 non-pregnant and non-breastfeeding female patients admitted for the evaluation of the hormonal function of adrenal incidentalomas. Anthropometric measurements, including body weight and height, were obtained, and the body mass index (BMI) of all participants was calculated. Glomerular filtration rate was estimated with CKD-EPI equation. An additional venous blood sample (7 mL) was collected from each patient to assess the concentrations of thyrotropin (TSH) and free thyroxine (fT_4_), as well as titers of anti-thyroid peroxidase (aTPO) and anti-thyroglobulin (aTG) antibodies. All analyses were performed on serum samples, withdrawn at the same day in fasting conditions. Serum samples were separated by centrifugation (3000 G) for 15 minutes and assessed using the Alinity Abbott system (Abbott Park, IL, USA). Thyroid ultrasonography was performed to evaluate the volume of both lobes, detect focal lesions, and identify features indicative of chronic thyroiditis. As the main part of the diagnostic work-up, the concentrations of iodine and creatinine were determined from a single morning spot urine sample and 24-hour urine collections.

### Iodine concentration measurements

The iodine concentration measurements in urine samples were performed using an Elan DRC-e (PerkinElmer Corp, USA/Sciex, Canada), ICP-MS (Inductively Coupled Plasma Mass Spectrometer) with an IsoMist (Glass Expansion, Australia) sample introduction system, featuring a thermally stabilized cyclonic spray chamber equipped with a MicroMist nebulizer. The matrix was modified with a TMAH (tetramethylammonium hydroxide) solution to achieve a concentration of 0.5% (w/v), which resulted in an alkaline pH of the samples, thereby preventing the formation of gaseous iodine compounds and reducing the memory effect.

Additional urinary iodine excretion parameters were calculated: iodine-to-creatinine ratio in a single morning urine spot sample (UI/CR) and iodine-to-creatinine ratio in a 24-hour urine collection (24-hour UI/CR).

### Data analysis

The currently recommended nutrient intake (RNI) of iodine by WHO is at least 150 μg for adults (except pregnant and lactating women), the same recommendations are valid in Poland (17). However, based on the analysis of urinary excretion and thyroidal accumulation in healthy subjects, 100 μg daily intake does not cause thyroid dysfunction related to iodine deficiency (18). This hypothesis-generating observation allows creation of a lower cut-off value to define the risk of iodine deficiency.

Therefore, we used two cutoff values for a 24-hour UIE: less than 100 μg to define the risk of deficiency, and at least 150 μg to define sufficiency (adequate iodine intake). For this study, we introduced the term ‘at-risk of iodine deficiency’ to refer to UIE values below 100 µg/day, which indicate an increased risk of insufficient thyroidal iodine uptake. In addition, we used the thresholds of iodine concentration in morning spot urine samples accepted by WHO for mild (50–99 μg/L), moderate (20-49 μg/L), and severe (< 20 μg/L) iodine deficiency (19).

According to WHO guidance, urinary iodine concentration cut-offs (mild/moderate/severe) are intended for assessing iodine status at the population level, and 24-hour urinary iodine excretion (24-h UIE), commonly treated as the individual-level reference measure in clinical/research contexts (20).

### Statistical analysis

Statistical analyses were performed using STATISTICA 13.0 PL (TIBCO Software Inc., Palo Alto, CA, USA), R software [R version 4.4.0 (2024-04–24 ucrt) – “Puppy Cup”, R Core Team (2021). R: A language and environment for statistical computing. R Foundation for Statistical Computing, Vienna, Austria]. A p-value of less than 0.05 determined statistical significance. All tests were two-tailed. Imputations were not performed for missing data. Nominal and ordinal data were expressed as numbers and percentages. Interval data were expressed as median, with lower (Q_1_) and upper (Q_3_) quartiles. The distribution of variables was evaluated by the Anderson-Darling test and the quantile-quantile (Q–Q) plot. Nominal and ordinal data were compared with the χ^2^ test or the Fisher test. Comparisons between groups for interval data were done with a non-parametric U Mann-Whitney test. We assessed the power of the tests not less than 80%. The cut-off value for deficiency in the 24-hour iodine excretion, based on iodine/creatinine in the urine spot sample, was assessed based on the ROC curve analysis. The ROC analysis was done with ‘*cut-point*’ and ‘*pROC*’ packages in R. The cut-off was established based on the maximization of the pooled mean from 2000 bootstrapped samples. The Youden index was used to assess the cut-off value. Results were presented as the area under the curve (AUC), accuracy (Acc), sensitivity (Se), specificity (Sp), positive and negative predictive values (PPV and NPV, respectively), as well as the diagnostic odds ratio, all with 95% confidence intervals (CI). The linear Pearson correlation coefficient and linear regression (with a 95% confidence interval and an ellipse of a multivariate t-distribution) were used to measure the associations between covariates.

## Results

### Study group characteristics

Only eight (16.3%) of the non-pregnant and non-breastfeeding women enrolled in this study had sufficient 24-hour UIE (at least 150 μg), as recommended (**Table 1**). The frequency of iodine deficiency at risk level (24-hour UIE <100 μg) in the study group was 55.1% (**Table 2**). Among these 27 subjects, only 6 had 24-hour UIE <60 μg/day (12.2% of the whole group). The lowest recorded 24-hour UIE was 27.4 μg.

**Table 1:** Characteristics of the study group and subgroups stratified into iodine sufficient and insufficient according to the 24-hour iodine urinary excretion threshold of 150 μg/day.

Variable	All subjects [N = 49]	Iodine sufficient [N = 8 (16.3%)]	Iodine insufficient [N = 41 (83.7%)]	P
Age [years]	65.0 (54.0 – 72.0)	61.5 (52.0 – 71.0)	67.0 (54.0 – 72.0)	0.84
BMI [kg/m^2^]	29.1 (25.1 – 31.7)	25.2 (23.8 – 28.6)	29.6 (26.0 – 32.1)	0.086
TSH [mIU/L]	1.31 (0.81 – 1.90)	1.05 (0.92 – 1.25)	1.46 (0.79 – 2.00)	0.16
fT_4_ [ng/dL]	0.97 (0.88 – 1.02)	0.94 (0.90 – 1.10)	0.97 (0.88 – 1.02)	0.75
Thyroid volume [mL]	10.8 (8.8 – 15.4)	12.4 (9.4 – 17.4)	10.7 (8.8 – 15.4)	0.38
eGFR [mL/min/1.73m^2^]	91.1 (83.1 – 95.1)	92.3 (86.9 – 93.1)	91.0 (78.4 – 96.9)	0.70
24-h iodine excretion [μg]	92.0 (70.4 – 131.5)	256.1 (163.6 – 324.9)	84.8 (65.7 – 104.9)	< 0.001
Iodine concentration in the spot sample [μg/L]	49.1 (32.3 – 73.8)	66.7 (40.3 – 95.8)	47.5 (31.7 – 63.9)	0.24
Above requirements ≥ 200 μg/L [N; %]	4; 8.2	4; 50.0	0	
Adequate 100-199 μg/L [N; %]	18; 36.7	4; 50.0	14; 34.1	
Mild deficiency 50-99 μg/L [N; %]	23; 46.9	0	23; 56.1	
Moderate deficiency – 20-49 μg/L [N; %]	4; 8.2	0	4; 9.8	
Iodine/creatinine in the urine spot sample [μg/g]	74.0 (57.0 – 100.0)	79.5 (66.0 – 145.0)	71.0 (54.2 – 93.0)	0.18
Creatinine concentration in the spot sample [mg/dL]	64.3 (40.0 – 105.0)	58.7 (39.8 – 108.0)	68.8 (40.0 – 105.0)	0.87
Iodine concentration in 24-h urine collection [μg/L]	42.3 (35.2 – 58.6)	96.6 (59.9 – 135.5)	41.1 (33.9 – 49.2)	< 0.001
24-h urinary iodine/creatinine [μg/g]	1.100 (0.820 – 1.470)	2.640 (1.510 – 3.300)	1.060 (0.770 – 1.290)	< 0.001
Creatinine concentration in 24-h urine collection [mg/dL]	40.9 (31.8 – 55.0)	41.5 (33.7 – 50.5)	40.9 (31.0 – 55.0)	0.86

Data presented as numbers with % or median with quartiles (Q_1_ – Q_3_).

**Table 2:** Electrolyte excretion in the study group and subgroups stratified into iodine sufficient and insufficient according to the 24-hour iodine urinary excretion threshold of 150 μg/day.

Variable	All subjects [N = 49]	Iodine sufficient [N = 8 (16.3%)]	Iodine insufficient [N = 41 (83.7%)]	P
24-h sodium excretion [mmol]	123.5 (106.3 – 171.9)	115.7 (97.7 – 152.7)	126.8 (106.3 – 175.0)	0.49
24-h sodium excretion < 87 mmol [N; %]	7; 14.3	2; 25.0	5; 12.2	0.35
24-h potassium excretion [mmol]	45.9 (36.0 – 62.0)	50.3 (42.1 – 59.9)	45.8 (34.3 – 62.4)	0.61
24-h urine collection volume [L]	2.2 (1.7 – 2.5)	2.6 (2.1 – 3.4)	2.1 (1.7 – 2.5)	0.071

Data presented as numbers with % or median with quartiles (Q_1_ – Q_3_).

The analysis of iodine concentration in the urinary spot sample showed mild deficiency in 23 women (46.9%) and moderate deficiency in 4 (8.2%) – **Table 1**. No cases of severe iodine deficiencies were observed in the analyzed group.

**Figure 1:**
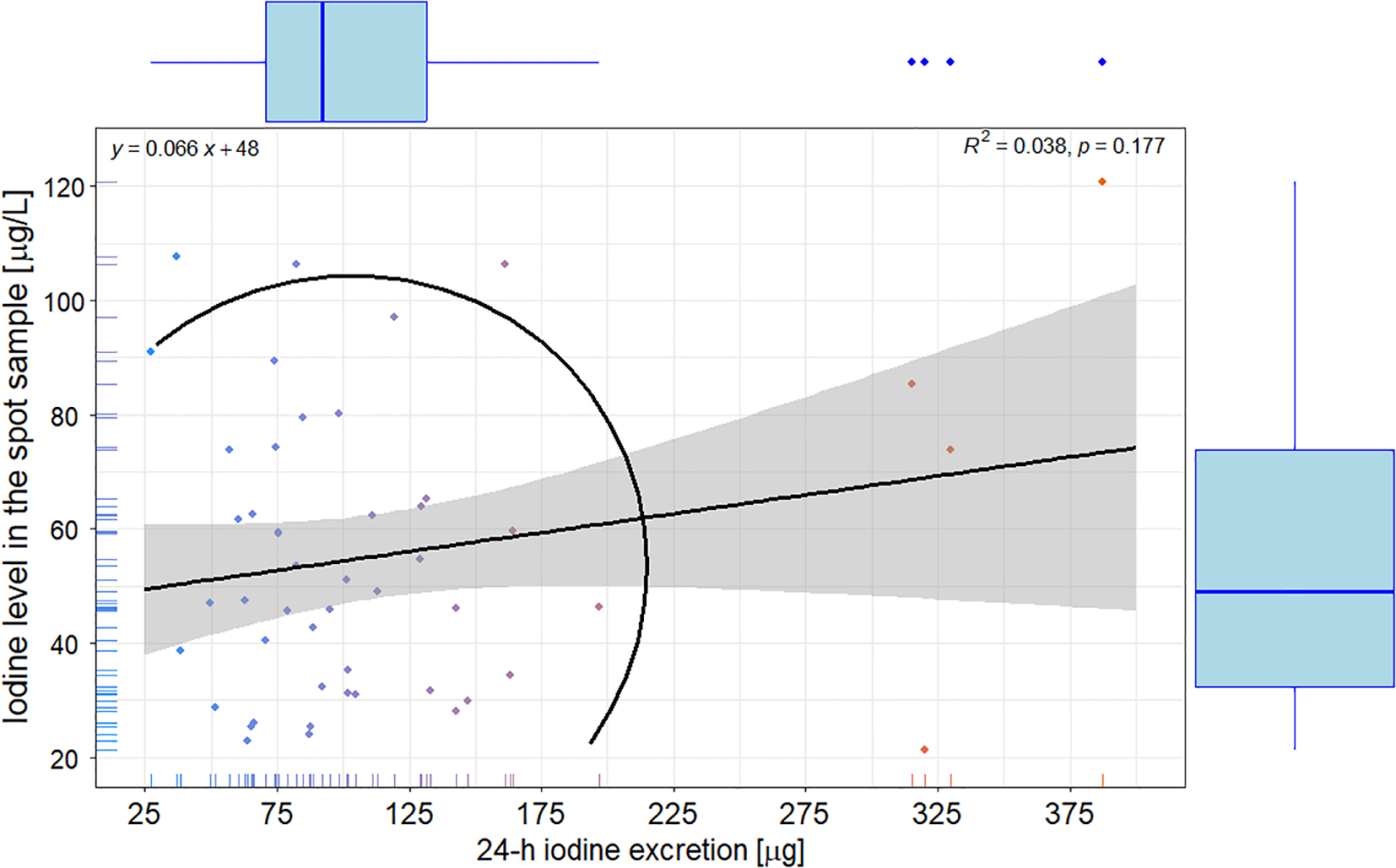
Scatterplot with correlation, linear regression with 95% confidence interval, and ellipse of multivariate t-distribution between the 24-h iodine urinary excretion and iodine concentration in the spot sample; color of points represents group with values of iodine-to-creatinine ratio ≤ and > 73 in the spot sample. Upper left corner: the linear regression equation; Upper right corner: R – the Pearson correlation coefficient.

Of note, there was no correlation between 24-hour UIE and iodine concentration in spot samples (R = 0.038; p = 0.172) – **Figure 1**.

Only 14.3% of all subjects had sodium excretion below 87 mmol/day, corresponding to the WHO recommendation of salt intake below 5 g/day – **Table 1**. None of the participants excreted less than 34 mmol of sodium (equivalent to 2 g of salt) in 24-hour urine collections.

### Iodine sufficiency and insufficiency

Among women with sufficient 24-hour UIE (at least 150 µg/day), half had urinary iodine concentrations within the WHO-recommended range. In contrast, the remaining half exceeded the upper reference limit based on spot urine samples (**Table 1**). Notably, 14 participants (34.1%) with suboptimal 24-hour UIE (below 150 µg/day) still demonstrated iodine concentrations in spot samples that fell within the recommended range. Kidney function, as assessed by estimated glomerular filtration rate (eGFR), as well as 24-hour urinary sodium and potassium excretion, did not differ significantly between the groups (**Table 2**).

### At-risk of iodine deficiency

The majority of subjects (85.2%) with 24-hour UIE <100 μg/day were classified as having mild iodine deficiency based on iodine concentration in morning spot urine samples. There was no association between iodine deficiency status and age, BMI, thyroid volume, or serum thyroid hormone levels. However, seropositivity for aTPO was more prevalent in the iodine-deficient group compared to subjects without deficiency (33.3% vs 8.0%; p < 0.05). Kidney function was similar in both subgroups (**Table 3**).

The iodine-deficient group had lower UI/Cr in the morning spot samples, regardless of comparable iodine concentrations.

**Figure 2:**
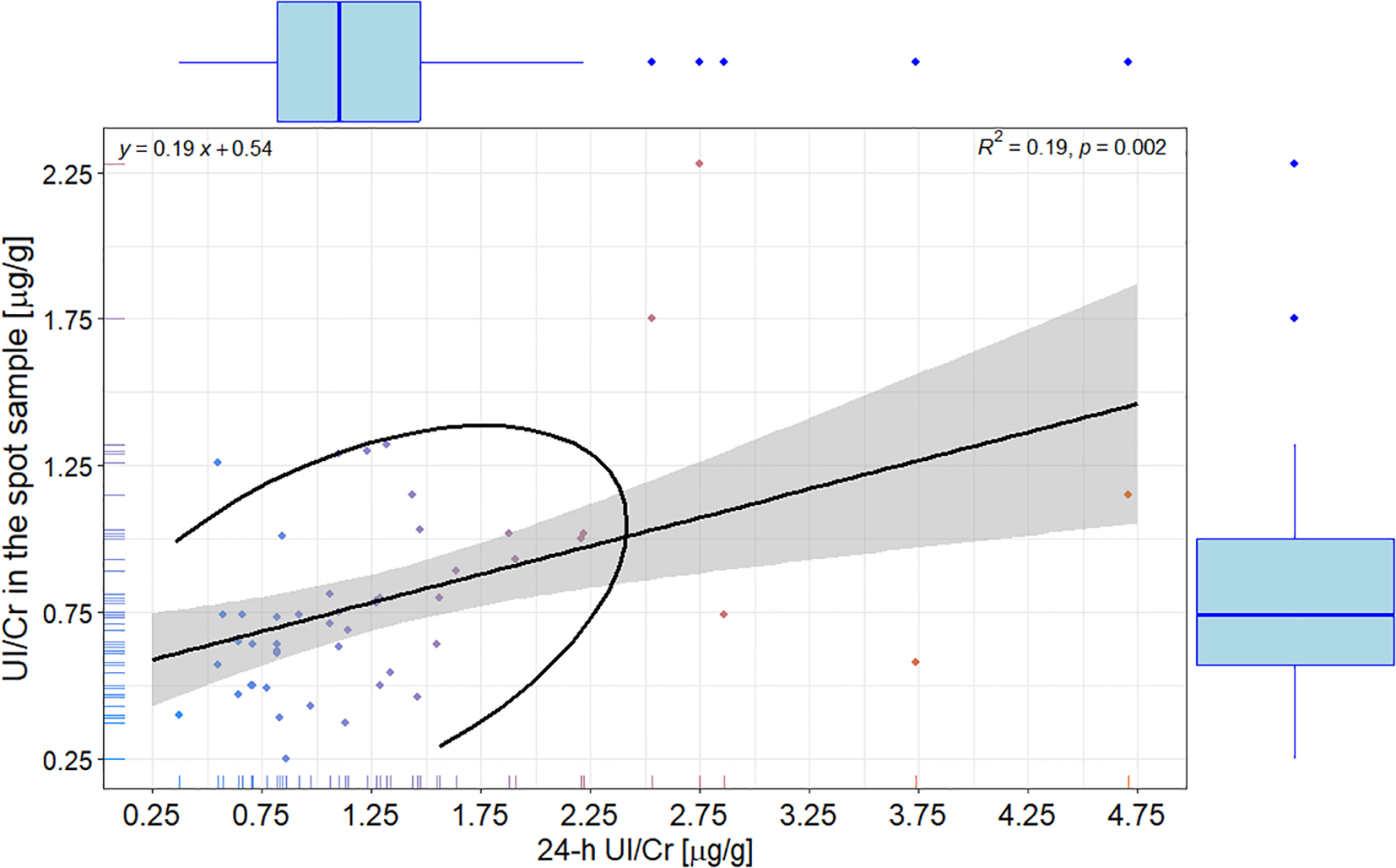
Scatterplot with correlation, linear regression with 95% confidence interval, and ellipse of multivariate t-distribution between the 24-h iodine-to-creatinine ratio and iodine-to-creatinine ratio in the spot sample; color of points represents group with values of iodine-to-creatinine ratio ≤ and > 73 in the spot sample. Upper left corner: the linear regression equation; Upper right corner: R – the Pearson correlation coefficient.

Notably, 24-hour UI/Cr were substantially higher than those derived from morning spot samples, with a significant, but weak correlation between the two (R = 0.196; R^2^ = 3.842%; p < 0.01) – **Figure 2**.

Participants with iodine deficiency exhibited lower daily sodium excretion; however, no correlation was found between iodine and sodium excretion (R = 0.102; R^2^ = 1.040%; p = 0.52).

### The iodine-to-creatinine ratios in the morning spot urine sample

**Figure 3:**
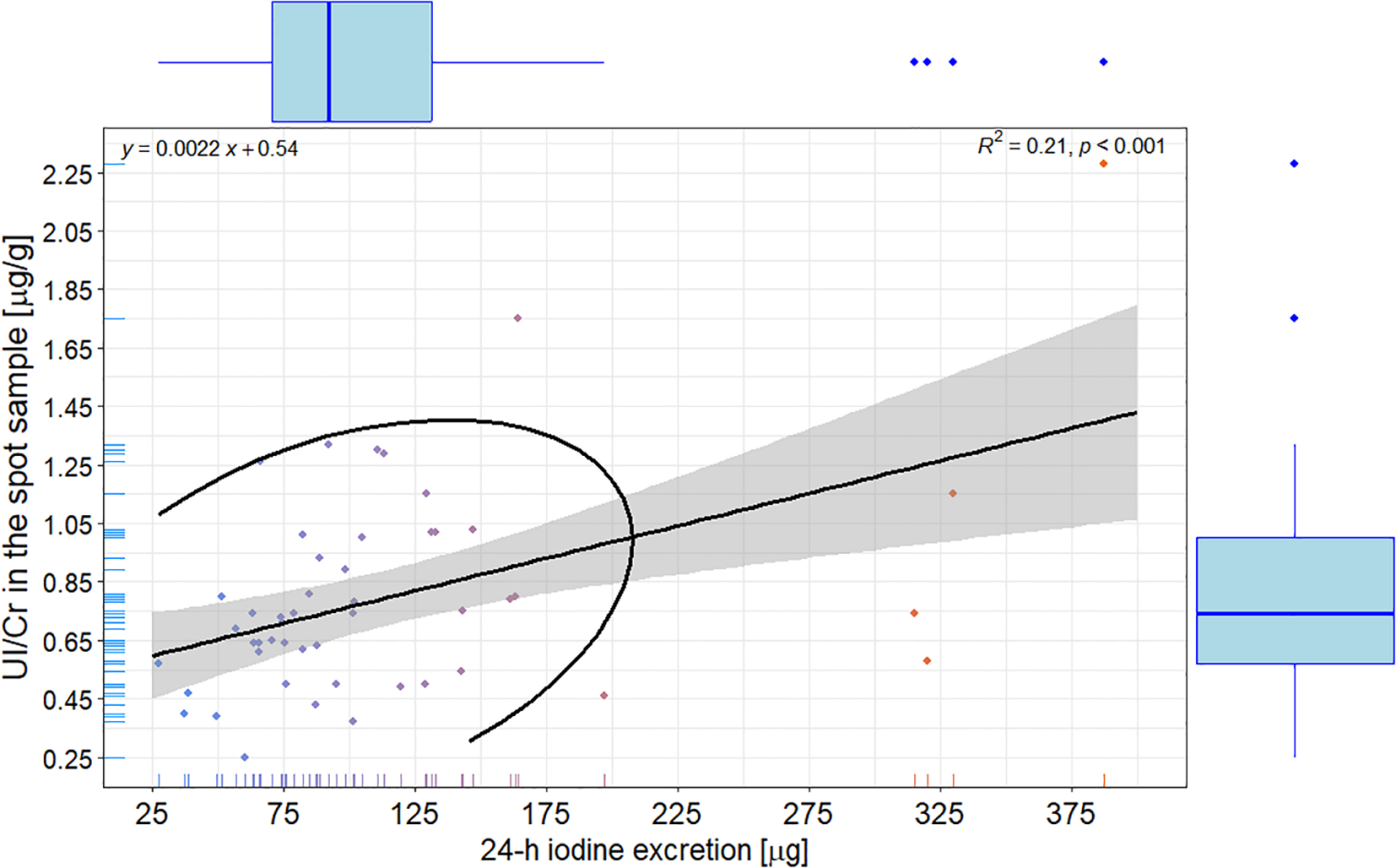
Scatterplot with correlation, linear regression with 95% confidence interval, and ellipse of multivariate t-distribution between the 24-h iodine excretion and iodine-to-creatinine ratio in the spot sample. Upper left corner: the linear regression equation; Upper right corner: R – the Pearson correlation coefficient.

There was a weak, but statistically significant correlation between 24-hour UIE and UI/Cr in the morning spot urine sample (R = 0.210; R^2^ = 4.410%; p < 0.001) – **Figure 3**.

**Figure 4:**
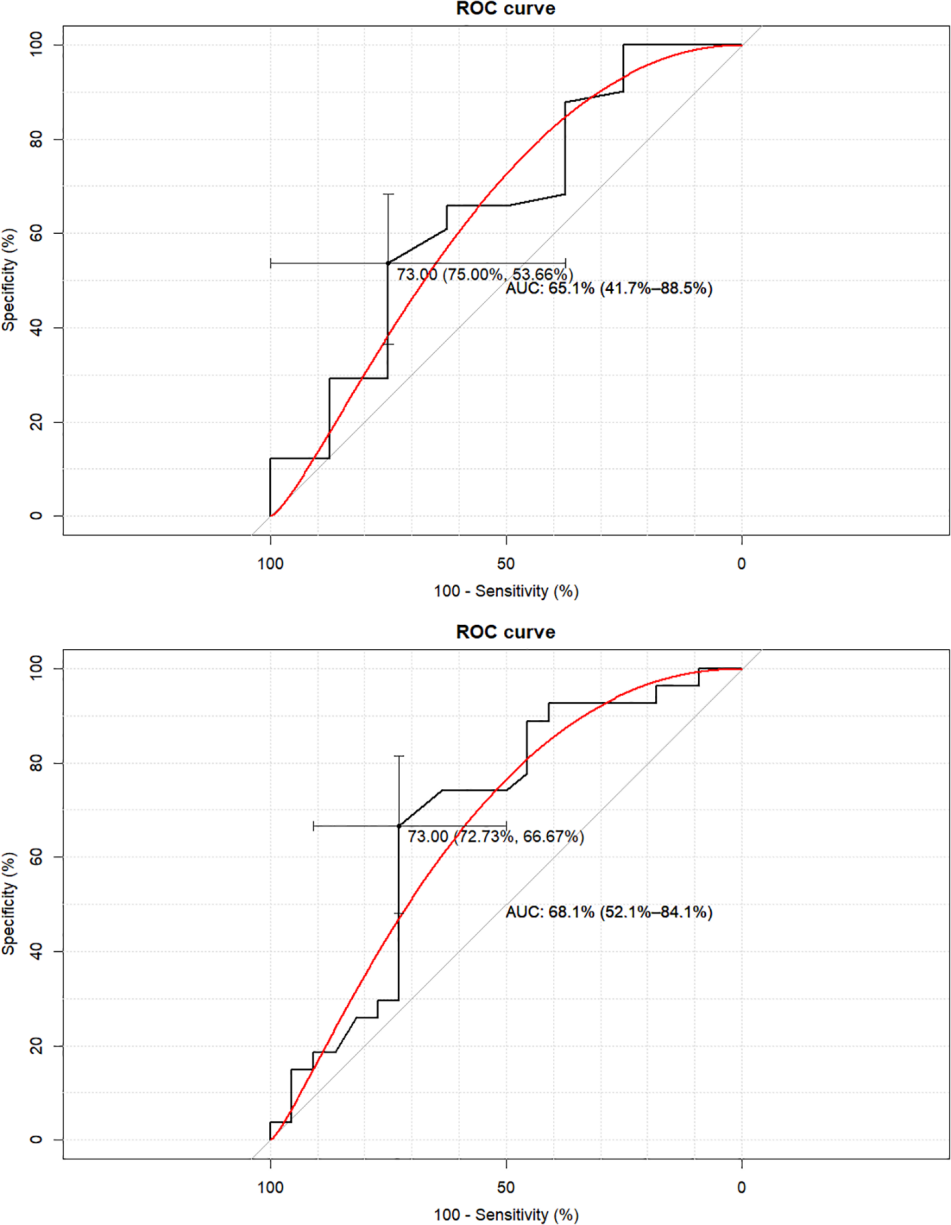
The ROC curves for iodine-to-creatinine ratio in the spot sample in the classification of 24-hour iodine excretion: sufficiency vs. non-sufficiency– threshold of 150 μg/day (upper panel), and deficiency vs. non-deficiency – threshold of 100 μg/day (lower panel). AUC, area under the curve; brackets represent 95% confidence interval.

The cut-off value for the UI/Cr in the morning spot urine samples discriminating iodine insufficiency, defined as 24-hour UIE < 150 μg, was established in ROC analysis at ≤ 73 μg/g. This threshold provided an accuracy of 57.1% (95% CI: 42.2 – 71.2%), with a sensitivity of 53.7% (95% CI: 37.4 – 69.3%), and a specificity of 75.0% (95% CI: 34.9 – 96.8%) – **Figure 4**. The corresponding positive and negative predictive values were 91.7% (95% CI: 73.0–99.0%) and 24.0% (95% CI: 9.4–45.1%), respectively. The diagnostic odds ratio was 3.47 (95% CI: 0.62 – 19.28).

**Table 3:** Characteristics of the study group and subgroups stratified into iodine-deficient and not iodine-deficient according to the 24-hour iodine urinary excretion threshold of 100 μg/day.

Variable	Not iodine-deficient [N = 22 (44.9%)]	Iodine deficient [N = 27 (55.1%)]	p
Age [years]	63.0 (54.0 – 71.0)	67.0 (52.0 – 72.0)	0.95
TSH [mIU/L]	1.23 (0.77 – 1.64)	1.37 (0.81 – 2.02)	0.21
fT_4_ [ng/dL]	0.99 (0.94 – 1.06)	0.95 (0.86 – 0.99)	0.07
Thyroid volume [mL]	11.2 (8.9 – 15.4)	10.6 (7.7 – 15.5)	0.38
eGFR [mL/min/1.73m^2^]	92.7 (86.1 – 95.1)	89.4 (74.7 – 97.3)	0.31
24-h iodine excretion [μg]	137.8 (112.9 – 164.1)	74.0 (60.3 – 84.8)	<0.001
Iodine concentration in the urine spot sample [μg/L]	50.0 (31.7 – 65.3)	47.5 (32.3 – 74.3)	0.90
Above requirements ≥ 200 μg/L [N; %]	4; 18.2	0	
Adequate 100-199 μg/L [N; %]	18; 81.2	0	
Mild deficiency 50-99 μg/L [N; %]	0	23; 85.2	
Moderate deficiency – 20-49 μg/L [N;%]	0	4; 14.8	
Iodine/creatinine in the urine spot sample [μg/g]	79.5 (58.0 – 115.0)	64.0 (50.0 – 80.0)	<0.05
Creatinine concentration in the urine spot sample [mg/dL]	54.2 (37.4 – 95.3)	90.5 (46.0 – 107.0)	0.12
Iodine concentration in 24-h urine collection [μg/L]	53.8 (47.5 – 79.1)	39.5 (31.5 – 42.0)	<0.001
24-h urinary iodine/creatinine [μg/g]	1.450 (1.230 – 2.220)	0.820 (0.660 – 1.100)	<0.001
Creatinine concentration in 24-h urine collection [mg/dL]	37.5 (31.8 – 49.0)	46.6 (30.4 – 55.0)	0.46

Data presented as numbers with % or median with quartiles (Q_1_ – Q_3_).

**Table 4:** Comparison of subgroups stratified based on urine iodine-to-creatinine ratio ≤ 73 (low) and > 73 (high) in spot sample.

Variable	UI/Cr > 73 [N = 25]	UI/Cr ≤ 73 [N = 22]	P
Age [years]	70.0 (56.0 – 72.0)	60.5 (49.5 – 69.0)	0.12
TSH [mIU/L]	1.31 (0.94 – 1.90)	1.33 (0.73 – 1.80)	0.90
fT_4_ [ng/dL]	0.97 (0.94 – 1.01)	0.95 (0.85 – 1.04)	0.39
Thyroid volume [mL]	11.1 (8.7 – 15.2)	10.7 (9.7 – 15.5)	0.76
eGFR [mL/min/1.73m^2^]	92.1 (83.2 – 94.6)	90.3 (77.5 – 96.1)	0.65
24-h iodine excretion [μg]	110.8 (88.7 – 147.1)	75.0 (62.0 – 98.2)	<0.01
Iodine concentration in spot sample [μg/L]	49.1 (31.7 – 73.9)	50.3 (37.0 – 68.3)	0.76
Excessive [N; %]	3; 12.0	1; 4.2	<0.05
Adequate [N; %]	13; 52.0	5; 20.8	
Mild deficiency [N; %]	9; 36.0	14; 58.3	
Moderate deficiency [N; %]	0	4; 16.7	
Iodine/creatinine in spot sample [μg/g]	100.0 (79.0 – 115.0)	55.6 (46.5 – 64.0)	<0.001
Creatinine concentration in spot sample [mg/dL]	47.9 (35.9 – 64.3)	98.2 (72.5 – 120.8)	<0.001
Iodine concentration in 24-h urine collection [μg/dL]	49.2 (40.8 – 63.3)	40.6 (33.6 – 47.6)	<0.05
24-h iodine/creatinine [μg/g]	1.320 (1.100 – 1.910)	0.825 (0.710 – 1.135)	<0.01
Creatinine concentration in 24-h urine collection [mg/dL]	37.5 (30.4 – 49.0)	44.2 (33.8 – 57.5)	0.18
24-h sodium excretion [mmol]	126.7 (108.9 – 182.3)	123.5 (105.4 – 155.8)	0.64

Data presented as numbers with % or median with quartiles (Q_1_ – Q_3_). UI/Cr = urine iodine to creatinine ratio.

A similar analysis was conducted to discriminate iodine deficiency, defined as 24-hour UIE < 100 μg/day, resulting in the same cut-off value of ≤ 73 μg/g. In this analysis, diagnostic accuracy was 69.4% (95% CI: 54.6 – 81.7%), with improved sensitivity of 66.7% (95% CI: 46.0 – 83.5%) and comparable specificity of 72.7% (95% CI: 49.8 – 89.3%) (**Figure 4**). The positive and negative predictive values for this cut-off were 75.0% (95% CI: 53.3 – 90.2%) and 64.0% (95% CI: 42.5 – 82.0%), respectively. The diagnostic odds ratio was 5.33 (95% CI: 1.55 – 18.30).

### Discriminating ability of iodine-to-creatinine ratio ≤ 73

The UI/Cr ratio cut-off of ≤ 73 correctly identified all 4 women with moderate iodine deficiency, indicating full discriminative ability in this subgroup. However, its utility was suboptimal for identifying mild iodine deficiency. Nine of 23 subjects (39.1%) with mild deficiency had UI/Cr ratio cut-off > 73. In addition, 6 of the 22 women (27.3%) without iodine deficiency would be incorrectly classified as deficient using this threshold (**Table 4**).

## Discussion

Our study showed that UI/Cr in the morning urine spot sample modestly reflects 24-h iodine excretion but is a more reliable surrogate measure than iodine concentration in the morning spot urine sample. We demonstrated no significant correlation between 24-hour UIE and iodine concentration in the morning spot urine sample (R = 0.038; p = 0.177), whereas a significant, albeit weak, correlation was observed with the UI/Cr (R = 0.210; p < 0.001). These findings are consistent with previous studies suggesting that spot UIC may substantially underestimate iodine intake due to its sensitivity to short-term dietary fluctuations and hydration status (9). However, it should be noted that measuring creatinine enables the adjustment for confounding physiological factors, such as fluid intake, urine concentrating ability, fasting state, time of sample collection, and physical activity (10). Moreover, the UI/Cr showed lower variability than urinary iodine concentration in spot samples, as shown among preschool children (20). Notably, UIC cut-offs proposed by the WHO are population-level metrics rather than individual diagnostic thresholds, which helps explain the observed discrepancies between spot measures (UIC, UI/Cr) and 24-hour UIE in our cohort. However, considering socioeconomic reasons, the collection of a single spot urine sample is more cost-effective and less burdensome than a 24-hour urine collection, which is time-consuming and prone to collection errors, potentially affecting the accuracy of the results (21).

The UI/Cr is beneficial for evaluating iodine status in cohorts with an increased risk of iodine deficiency, such as pregnant and breastfeeding women, as well as children. These groups have greater variability in iodine requirements and intake. Moreover, fluctuations in fluid balance are more prevalent in these populations, increasing the likelihood of urine dilution, especially during pregnancy (10, 22).

Our findings reveal a high prevalence of iodine insufficiency and deficiency in non-pregnant and non-breastfeeding women. Only 16.3% met the recommended threshold of ≥ 150 µg/day, and more than half of the participants had a 24-hour UIE below 100 µg/day. Despite this, the diagnostic performance of the UI/Cr was suboptimal for detecting mild insufficiency, with sensitivity and specificity values of 53.7% and 75.0%, respectively, and a diagnostic odds ratio of 3.47. However, it showed better performance in identifying iodine deficiency (< 100 µg/day), with improved sensitivity (66.7%) and diagnostic odds ratio (5.33). These, as well as higher UI/Cr in 24-hour urine collections than in spot urinary samples, may correspond to a non-linear relationship between daily iodine intake and daily iodine retention. Notably, all participants with moderate iodine deficiency were correctly classified, suggesting potential utility in identifying more severely deficient cases.

The recommended daily iodine intake of 150 µg for adult non-pregnant and non-breastfeeding women, established more than 50 years ago, may be considered excessive. It was recently shown that balanced iodine excretion can be achieved with a daily iodine intake of 63.4 μg in males, corresponding to 61.6 µg in females (23). Although lower than the recommended daily iodine intake, it might be considered sufficient to prevent iodine deficiency. Of note, in our study group, only 12.2% of subjects had 24-hour urine excretion < 60 µg. Possibly only this group should be considered at risk of iodine deficiency. The significance of these findings is essential in the context of the increasing incidence of thyroid cancer, partially attributed to increased iodine intake, worldwide (24, 25).

A weak, but significant correlation between iodine and sodium excretion in 24-hour urine collection suggests that dietary salt intake may not be the primary source of iodine in the diet. Therefore, further restrictions on salt intake, as recommended by the WHO and cardiology societies, may not necessarily exacerbate iodine deficiency. It is possible that other iodine sources, such as seafood, eggs, dairy products, and certain types of fish, are becoming increasingly significant. Among plant-based sources, seaweed and algae are known to be rich in iodine; nevertheless, they are not commonly consumed in Western diets. Notably, the soil mineral composition also affects the iodine content of plants. The vegetarian and, even more so, vegan diets are associated with an increased risk of iodine insufficiency (26). Although previously published studies indicate that iodized salt is the primary source of iodine in the Polish population, our findings do not align with this observation (27, 28). This may reflect changing dietary habits and the growing popularity of Eastern diets. Furthermore, potential gender-related dietary differences should also be considered. There is lack of current research concerning the main sources of iodine intake in the Polish population.

Interestingly, seropositivity for anti-thyroid peroxidase antibodies (aTPO) was more prevalent in the iodine-deficient group. However, this association should be interpreted cautiously due to the lack of data on iodine consumption during childhood and adolescence, as well as the small sample size. These data are insufficient to raise questions about the role of chronic iodine deficiency in the development of thyroid autoimmunity. It should also be noted that previous studies more commonly link excessive iodine exposure to autoimmune thyroiditis (29, 30).

### Study limitations

The primary limitation of this study is the evaluation of iodine excretion under hospital dietary conditions, which may not reflect participants’ usual dietary habits. While this may affect the evaluation of iodine sufficiency and deficiency, the controlled hospital setting likely improved the completeness and accuracy of 24-hour urine collection, which is crucial for a reliable measurement of iodine excretion. The second limitation is the small sample size, a consequence of the strict exclusion criteria applied to hospitalized patients. Finally, the findings may not be fully generalizable to older old, pregnant and breastfeeding women, and male subjects concerning differences in eating habits.

## Conclusions

1. Iodine to creatine ratio in morning spot urine sample modestly reflects 24-hour iodine urinary excretion, but more accurately than iodine concentration alone.
2. The cut-off value of 73 μg/g for the iodine-to-creatinine ratio in morning spot urine samples offers moderate diagnostic performance in identifying iodine deficiency.
3. A daily iodine intake of 100 µg appears sufficient to prevent deficiency in non-pregnant and non-breastfeeding women, indicating a need to reconsider current recommendations for iodine intake. Although our findings suggest that some individuals maintain adequate thyroid function at intakes around 100 µg/day, larger studies are necessary to confirm whether such lower-than-recommended levels can consistently support thyroid health without increasing the risk of deficiency.

## Data Availability

The raw data supporting the conclusions of this article will be made available by the authors, without undue reservation.

## References

[1] Winder M Kosztyła Z Boral A Kocełak P Chudek J . The impact of iodine concentration disorders on health and cancer. Nutrients. (2022) 14:2209. doi: 10.3390/nu14112209, PMID: 35684009 PMC9182735

[2] Rayman MP Bath SC . The new emergence of iodine deficiency in the UK: consequences for child neurodevelopment. Ann Clin Biochem. (2015) 52:705–8. doi: 10.1177/0004563215597249, PMID: 26240435

[3] Zimmermann MB Andersson M . GLOBAL ENDOCRINOLOGY: Global perspectives in endocrinology: coverage of iodized salt programs and iodine status in 2020. Eur J Endocrinol. (2021) 185:R13–21. doi: 10.1530/eje-21-0171, PMID: 33989173 PMC8240726

[4] Haap M Roth HJ Huber T Dittmann H Wahl R . Urinary iodine: comparison of a simple method for its determination in microplates with measurement by inductively-coupled plasma mass spectrometry. Sci Rep. (2017) 7:39835. doi: 10.1038/srep39835, PMID: 28045077 PMC5206638

[5] WHO Secretariat Andersson M de Benoist B Delange F Zupan J . Prevention and control of iodine deficiency in pregnant and lactating women and in children less than 2-years-old: conclusions and recommendations of the Technical Consultation. Public Health Nutr. (2007) 10:1606–11. doi: 10.1017/s1368980007361004, PMID: 18053287

[6] Ittermann T Nautsch A Schmidt CO Kramer A Below H Remer T . High (but not low) urinary iodine excretion is predicted by iodine excretion levels from five years ago. Ann Nutr Metab. (2011) 58:335–42. doi: 10.1159/000331991, PMID: 21985800

[7] Bu Y Cai Y Ji C Zhao C Tian C Pang B . Evaluation of iodine nutritional status during pregnancy by estimated 24-h urinary iodine excretion: population variation range and individual accuracy. Public Health Nutr. (2022) 25:237–47. doi: 10.1017/s1368980021003335, PMID: 34380579 PMC8883787

[8] Li C Peng S Zhang X Xie X Wang D Mao J . The urine iodine to creatinine as an optimal index of iodine during pregnancy in an iodine adequate area in China. J Clin Endocrinol Metab. (2016) 101:1290–8. doi: 10.1210/jc.2015-3519, PMID: 26789777

[9] Rasmussen LB Ovesen L Christiansen E . Day-to-day and within-day variation in urinary iodine excretion. Eur J Clin Nutr. (1999) 53:401–7. doi: 10.1038/sj.ejcn.1600762, PMID: 10369497

[10] Oblak A Hribar M Hristov H Gregorič M Blaznik U Osredkar J . Interpreting urinary iodine concentration: effects of urine dilution and collection timing. Eur J Clin Nutr. (2024) 78:1105–10. doi: 10.1038/s41430-024-01492-y, PMID: 39117906 PMC11611732

[11] Haddow JE McClain MR Palomaki GE Hollowell JG . Urine iodine measurements, creatinine adjustment, and thyroid deficiency in an adult United States population. J Clin Endocrinol Metab. (2007) 92:1019–22. doi: 10.1210/jc.2006-2156, PMID: 17200163

[12] Bu Y Yuan L Tian C Zhao C Ji C Gao X . 24 h urinary creatinine excretion during pregnancy and its application in appropriate estimation of 24 h urinary iodine excretion. J Trace Elem Med Biol. (2021) 66:126751. doi: 10.1016/j.jtemb.2021.126751, PMID: 33836494

[13] Dahl L Wik Markhus M Sanchez PVR Moe V Smith L Meltzer HM . Iodine deficiency in a study population of norwegian pregnant women-results from the little in Norway study (LiN). Nutrients. (2018) 10:513. doi: 10.3390/nu10040513, PMID: 29677112 PMC5946298

[14] Fuse Y Ito Y Tsukada N Shishiba Y Irie M . Iodine intake in healthy Japanese aged from 6 to 70 years residing in the same district. Endocr J. (2022) 69:253–62. doi: 10.1507/endocrj.ej21-0479, PMID: 34602518

[15] König F Andersson M Hotz K Aeberli I Zimmermann MB . Ten repeat collections for urinary iodine from spot samples or 24-hour samples are needed to reliably estimate individual iodine status in women. J Nutr. (2011) 141:2049–54. doi: 10.3945/jn.111.144071, PMID: 21918061

[16] World Health Organisation . Urinary iodine concentrations for determining iodine status deficiency in populations. In: Vitamin and Mineral Nutrition Information System. World Health Organization, Geneva (2013). Available online at: http://www.who.int/nutrition/vmnis/indicators/urinaryiodine (Accessed November 14, 2025).

[17] Jarosz M Rychlik E Stoś K Charzewska J . Normy żywienia dla populacji polskiej i ich zastosowania. Narodowy Instytut Zdrowia Publicznego –Państwowy Zakład Higieny. (2020), 293–5.

[18] Vought RL London WT . Iodine intake, excretion and thyroidal accumulation in healthy subjects. J Clin Endocrinol Metab. (1967) 27:913–9. doi: 10.1210/jcem-27-7-913, PMID: 4165697

[19] World Health Organization International Council for the Control of the Iodine Deficiency Disorders United Nations Children’s Fund . Assessment of the Iodine Deficiency Disorders and Monitoring Their Elimination. Geneva, Switzerland: World Health Organization (2007).

[20] Soldin OP . Controversies in urinary iodine determinations. Clin Biochem. (2002) 35:575–9. doi: 10.1016/S0009-9120(02)00406-X, PMID: 12498990 PMC3637997

[21] Dong A Rui Y Yuping D Xuan W Ying Y Wenxing G . Variations in the urinary iodine concentration and urinary iodine/creatinine ratio among preschool children. Int J Endocrinol. (2023) 6779094. doi: 10.1155/2023/6779094

[22] Vejbjerg P Knudsen N Perrild H Laurberg P Andersen S Rasmussen LB . Estimation of iodine intake from various urinary iodine measurements in population studies. Thyroid. (2009) 19:1281–6. doi: 10.1089/thy.2009.0094, PMID: 19888863

[23] Yang L Wang J Yang J Zhang H Liu X Mao D . An iodine balance study to explore the recommended nutrient intake of iodine in Chinese young adults. Br J Nutr. (2020) 124:1156–65. doi: 10.1017/s0007114520002196, PMID: 32624007

[24] Huang F Cong W Xiao J Zhou Y Gong M Sun J . Association between excessive chronic iodine exposure and the occurrence of papillary thyroid carcinoma. Oncol Lett. (2020) 20:189. doi: 10.3892/ol.2020.12051, PMID: 32952658 PMC7479532

[25] Lv C Gao Y Yao J Li Y Lou Q Zhang M . High iodine induces the proliferation of papillary and anaplastic thyroid cancer cells via AKT/wee1/CDK1 axis. Front Oncol. (2021) 11:622085. doi: 10.3389/fonc.2021.622085, PMID: 33796458 PMC8008130

[26] Rogerson D . Vegan diets: practical advice for athletes and exercisers. J Int Soc Sports Nutr. (2017) 14:36. doi: 10.1186/s12970-017-0192-9, PMID: 28924423 PMC5598028

[27] Krela-Kaźmierczak I Czarnywojtek A Skoracka K Rychter AM Ratajczak AE Szymczak-Tomczak A . Is there an ideal diet to protect against iodine deficiency? Nutrients. (2021) 13:513. doi: 10.3390/nu13020513, PMID: 33557336 PMC7914421

[28] Zaremba A Gramza-Michalowska A Pal K Szymandera-Buszka K . The effect of a vegan diet on the coverage of the recommended dietary allowance (RDA) for iodine among people from Poland. Nutrients. (2023) 15:1163. doi: 10.3390/nu15051163, PMID: 36904161 PMC10005417

[29] Xu C Wu F Mao C Wang X Zheng T Bu L . Excess iodine promotes apoptosis of thyroid follicular epithelial cells by inducing autophagy suppression and is associated with Hashimoto thyroiditis disease. J Autoimmun. (2016) 75:50–7. doi: 10.1016/j.jaut.2016.07.008, PMID: 27448770

[30] Gong B Meng F Wang X Han Y Yang W Wang C . Effects of iodine intake on gut microbiota and gut metabolites in Hashimoto thyroiditis-diseased humans and mice. Commun Biol. (2024) 7:136. doi: 10.1038/s42003-024-05813-6, PMID: 38287080 PMC10824742

